# Impaired Expression of Ectonucleotidases in Ectopic and Eutopic Endometrial Tissue Is in Favor of ATP Accumulation in the Tissue Microenvironment in Endometriosis

**DOI:** 10.3390/ijms20225532

**Published:** 2019-11-06

**Authors:** Carla Trapero, August Vidal, Maria Eulàlia Fernández-Montolí, Buenaventura Coroleu, Francesc Tresserra, Pere Barri, Inmaculada Gómez de Aranda, Jean Sévigny, Jordi Ponce, Xavier Matias-Guiu, Mireia Martín-Satué

**Affiliations:** 1 Departament de Patologia i Terapèutica Experimental, Facultat de Medicina i Ciències de la Salut, Campus Bellvitge, Universitat de Barcelona, 08907 Barcelona, Spain; ctrapero@idibell.cat (C.T.); avidal@bellvitgehospital.cat (A.V.); igomezdearanda@ub.edu (I.G.d.A.); 2 Oncobell Program, CIBERONC, Institut d’Investigació Biomèdica de Bellvitge (IDIBELL), 08908 Barcelona, Spain; mefernandez@bellvitgehospital.cat (M.E.F.-M.); jponce@bellvitgehospital.cat (J.P.); fjmatiasguiu.lleida.ics@gencat.cat (X.M.-G.); 3 Servei d’Anatomia Patològica, Hospital Universitari de Bellvitge, 08907 Barcelona, Spain; 4 Servei de Ginecologia, Hospital Universitari de Bellvitge, 08907 Barcelona, Spain; 5 Salud de la Mujer Dexeus, Hospital Universitari Quiron Dexeus, 08028 Barcelona, Spain; VENCOR@dexeus.com (B.C.); francesc.tresserra@quironsalud.es (F.T.); barper@dexeus.com (P.B.); 6 Centre de Recherche du CHU de Québec - Université Laval, Québec City, QC G1V 4G2, Canada; jean.sevigny@crchudequebec.ulaval.ca; 7 Départment de Microbiologie-Infectiologie et d’Immunologie, Faculté de Médecine, Université Laval, Quebec City, QC G1V 4G2, Canada

**Keywords:** endometriosis, endometrium, uterus, purinergic signaling, ATP

## Abstract

Endometriosis is a prevalent disease defined by the presence of endometrial tissue outside the uterus. Adenosine triphosphate (ATP), as a proinflammatory molecule, promotes and helps maintain the inflammatory state of endometriosis. Moreover, ATP has a direct influence on the two main symptoms of endometriosis: infertility and pain. Purinergic signaling, the group of biological responses to extracellular nucleotides such as ATP and nucleosides such as adenosine, is involved in the biology of reproduction and is impaired in pathologies with an inflammatory component such as endometriosis. We have previously demonstrated that ectonucleotidases, the enzymes regulating extracellular ATP levels, are active in non-pathological endometria, with hormone-dependent changes in expression throughout the cycle. In the present study we have focused on the expression of ectonucleotidases by means of immunohistochemistry and in situ activity in eutopic and ectopic endometrial tissue of women with endometriosis, and we compared the results with endometria of women without the disease. We have demonstrated that the axis CD39-CD73 is altered in endometriosis, with loss of CD39 and CD73 expression in deep infiltrating endometriosis, the most severe, and most recurring, endometriosis subtype. Our results indicate that this altered expression of ectonucleotidases in endometriosis boosts ATP accumulation in the tissue microenvironment. An important finding is the identification of the nucleotide pyrophophatase/phosphodiesterase 3 (NPP3) as a new histopathological marker of the disease since we have demonstrated its expression in the stroma only in endometriosis, in both eutopic and ectopic tissue. Therefore, targeting the proteins directly involved in ATP breakdown could be an appropriate approach to consider in the treatment of endometriosis.

## 1. Introduction

Endometriosis is a chronic gynecological estrogen-dependent disease characterized by the presence of endometrial tissue, both glands and stroma, outside the uterus. There are multiple possible locations for this ectopic tissue which may be grouped into three endometriosis subtypes: peritoneal, ovarian, with ovarian cysts called endometriomas, and deeply infiltrative. It is a debilitating disorder affecting around 10% of women of reproductive age [1], with pelvic pain and infertility as the two main symptoms. The etiology and physiopathology of this disease remain unknown and there are no clinical biomarkers; in consequence, there is no cure and a long delay in the diagnosis. Studies focused on the discovery of diagnostic tools for endometriosis to help to design effective treatments that preserve fertility are of great interest.

Inflammation is necessary for the establishment and maintenance of endometrial cells in ectopic locations [2,3,4,5]. Purinergic signaling, the group of biological effects mediated by extracellular nucleotides, such as adenosine triphosphate (ATP), and nucleosides, such as adenosine, is involved in a wide range of physiological and pathological inflammatory conditions [6]. Extracellular ATP is mostly a proinflammatory molecule released during tissue stress situations, such as necrosis or apoptosis, hypoxia, and inflammation. Purinergic signaling is also studied in the context of human reproduction [7,8,9,10,11]; for instance, ATP is involved in the initiation and maintenance of myometrium and oviduct contractions. It increases the oviductal ciliary beat frequency [12] and contributes to the regulation of the uterine fluid microenvironment [9]. Moreover, adenosine, an ATP hydrolysis product, is necessary for sperm capacitation [10]. ATP is also a pain-related molecule, and some of the pharmacological treatments used to relieve pain in endometriosis do indeed affect ATP levels or their effects. Moreover, extracellular ATP and its derivative adenosine influence cell migration, proliferation and survival—three necessary events for the establishment of ectopic endometrial foci.

Extracellular ATP and adenosine levels are controlled by the ecto-nucleotidases, which are broadly expressed enzymes that, acting alone or sequentially, hydrolyze ATP into adenosine. There are four families of ectonucleotidases: (i) the ecto-nucleoside triphosphate diphosphohydrolase (E-NTPDase) family, also known as CD39 family, which hydrolyzes the ATP, and adenosine diphosphate (ADP), to adenosine monophosphate (AMP); (ii) the ecto-nucleotide pyrophophatase/phosphodiesterase (E-NPP) family, which mainly hydrolyzes ATP to AMP; (iii) the 5′-nucleotidase (5′-NT) (known as CD73) that dephosphorylates AMP to adenosine; and, (iv) the alkaline phosphatases family that hydrolyzes nucleoside triphosphates and diphosphates to monophosphates [6]. Adenosine deaminase (ADA) inactivates adenosine. This is a soluble enzyme often associated with CD26/dipeptidyl peptidase IV, expressed at the cell membrane [13]. Ectonucleotidases and CD26 are well characterized in human cyclic and postmenopausal endometria, showing differences in its expression and distribution throughout the cycle [8,14]. The presence of ectonucleotidases in the contents of endometrioma had previously been described [15,16] but no studies had yet to be conducted on eutopic and ectopic endometrial tissue from women with endometriosis.

With the present study, we aimed to characterize the expression of ectonucleotidases in the eutopic and ectopic endometrial tissue of women with endometriosis and compare it with the eutopic endometrium of women without this pathology. We believe that assessing the participation of these proteins directly involved in ATP breakdown in endometriosis could contribute to facilitating the diagnosis and ameliorating the treatment status of this pathology.

## 2. Results

Protein expression of NTPDase1 (CD39 from here on), NTPDase2, NTPDase3, NPP3, 5′-NT (CD73 from here on), and CD26 was detected in the eutopic and ectopic endometrial tissue of women with endometriosis. Results are compared with endometria from women without the disease whose data was previously published [8,14]. Staining distribution and intensity scores were recorded for each protein by double blinded observation. In situ nucleotidase activity, in the presence or absence of specific inhibitors, was also detected. The most relevant findings in eutopic endometria and endometrial lesions are described for each protein hereafter, and compiled in detail in Table 1 and Table 2. The proportion of positive tissues stained in the immunolabeling assays is commented on in the text and compiled in Appendix A (Table A1).

### 2.1. CD39 Expression in the Eutopic and Ectopic Endometrial Tissues

**Figure 1:**
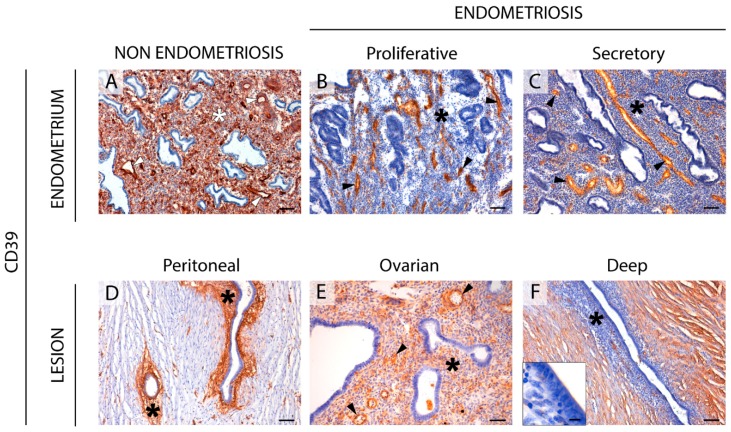
Immunolocalization of CD39 in eutopic (**A**–**C**) and ectopic (**D**–**F**) endometrial tissue. CD39 was expressed in blood vessels (arrowheads) of eutopic endometria from women without (NON ENDOMETRIOSIS, (**A**) or with endometriosis (ENDOMETRIOSIS, (**B**,**C**), and in peritoneal (**D**), ovarian (**E**), and deep infiltrating (**F**) lesions. CD39 was immunodetected in the stroma (asterisk) of endometrium of women without the disease (**A**), but not in endometria of women with endometriosis (**B**,**C**). Stroma is also labelled in the peritoneal (**D**) and ovarian (**E**) ectopic tissues of women with endometriosis. Inset in (**F**) is a detail of a deep lesion (oviductal infiltrating nodule) showing the absence of CD39 in the endometrial stroma. Scale bars are 100 μm (**A**–**F**) and 10 μm (**F** inset).

CD39 staining was detected in stromal cells and in endothelial cells from blood vessels of endometria from women without endometriosis (Figure 1), coinciding with previously published findings [8]. In the eutopic cyclic endometria from women with endometriosis, CD39 label in the stroma was absent in 65% of cases although it was always present in endothelial cells (Figure 1). However, atrophic endometria from women with endometriosis maintained the CD39 expression in the stromal component (Figure S1).

**Figure 2:**
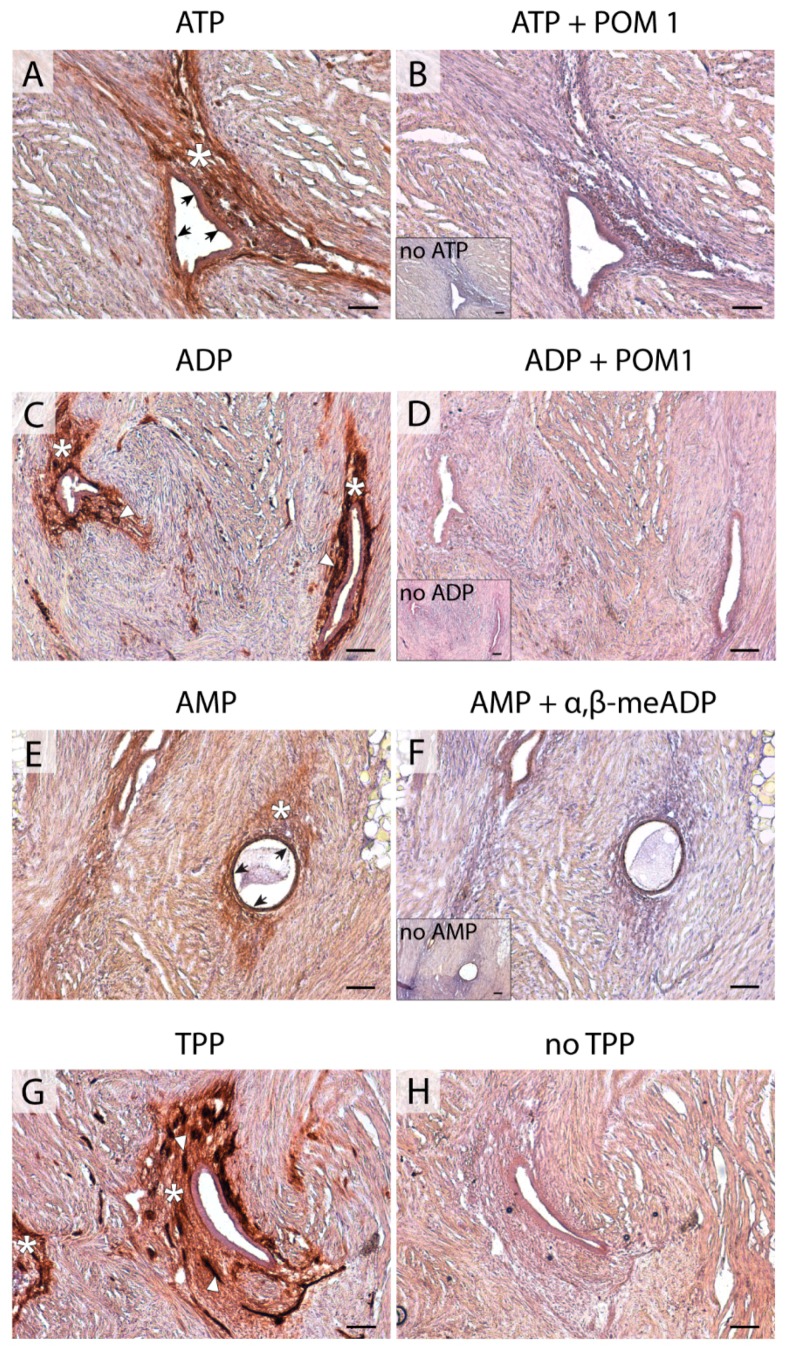
Nucleotidase in situ histochemistry in superficial peritoneal endometriosis lesions. Dark brown deposits correspond to enzyme activity. ATPase activity was detected in the epithelial cells (arrows) and stroma (asterisk) of the lesion (**A**). ADPase activity was strongly detected in the stroma (asterisks) of the lesion and, remarkably, in blood vessels (arrowheads) (**C**). ATPase and ADPase activities were abrogated in the presence of the NTPDase inhibitor POM 1 (**B**,**D**, respectively). In situ AMPase activity was detected in the epithelial (arrows) and stromal (asterisk) components of the lesion (**E**). The AMPase was inhibited in the presence of α,β-meADP (**F**). Insets in (**B**,**D**,**F**) correspond to the activity experiments performed in the absence of substrate (no ATP, no ADP, and no AMP, respectively). TPPase activity was distributed in the stroma (asterisks), including the blood vessels (arrowheads) of lesions (**G**). As a control, TPPase activity was also performed without substrate (no TPP, **H**). Scale bars are 100 μm.

In endometriotic lesions, CD39 was immunodetected with strong labeling in the stroma of the peritoneal (86% of cases) and ovarian (59%) lesions, but not in the deep ones, where this label was only in 36% of the samples. Moreover, 54% of deeply infiltrative lesions lost the expression of CD39 in blood vessels. ADPase activity was seen at the same locations where the protein was immunodetected. The activity was inhibited by the E-NTPDase inhibitor POM 1 (Figure 2).

### 2.2. NTPDase2 Expression in the Eutopic and Ectopic Endometrial Tissues

**Figure 3:**
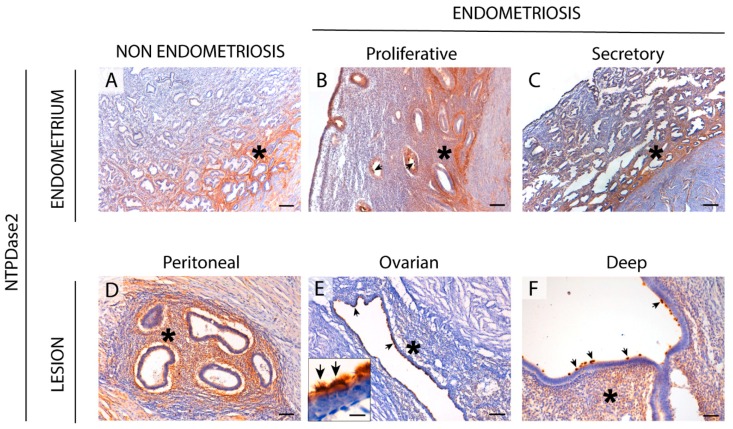
Immunolocalization of NTPDase2 in the eutopic (**A**–**C**) and ectopic (**D**–**F**) endometrial tissue. NTPDase2 was detected in the stroma of the basal layer (asterisks) in cyclic endometria from women without (**A**) and with (**B**,**C**) endometriosis. It was also present in the stromal component (asterisks) of the superficial peritoneal (**D**) and deep infiltrating lesions (**F**, vaginal nodule). NTPDase2 was also found in eutopic and ectopic epithelial ciliated cells in the cilia (arrows) (detail in the inset in **E**). Scale bars are 200 μm (**A**,**C**), 100 μm (**B**,**D**–**F**), and 10 μm (inset in **E**).

NTPDase2 label in endometria of women with endometriosis was found in basal stroma and cilia of epithelial ciliated cells (Figure 3), coinciding with the recently described expression in non-pathological endometria [14]. All types of ectopic lesions displayed cilia staining, except three deeply infiltrative lesions localized in oviducts that did not display NTPDase2 staining in the cilia. However, the rest of the deep lesions with another ectopic location showed NTPDase2 labeling in their ciliated cells. Stroma was labeled in peritoneal (100% of the cases) and deep lesions (65%), but much less so in ovarian lesions (37%). In situ ATPase activity was detected in the same locations where NTPDase2 was expressed, and it was inhibited by the E-NTPDase inhibitor POM 1 (Figure 2).

Interestingly, we observed the NTPDase2 label in the connective tissue that surrounds the lesions clearly defining the limits of the ectopically located endometrial tissue in a large number of cases (Figure S2).

Perivascular NTPDase2+ cells were also found in eutopic and ectopic endometrial tissue. We confirmed that these cells were also positive for the endometrial mesenchymal stem cell (eMSC) marker Sushi Domain Containing 2 (SUSD2). Double immunolabeling for NTPDase2 and SUSD2 revealed that both proteins were expressed by the same perivascular cells (Figure S3). This coincides with recently published results in non-pathological endometria [14]. Nevertheless, NTPDase2+ SUSD2+ perivascular cells were not present in all the endometriotic lesions.

### 2.3. NTPDase3 Expression in the Eutopic and Ectopic Endometrial Tissues

**Figure 4:**
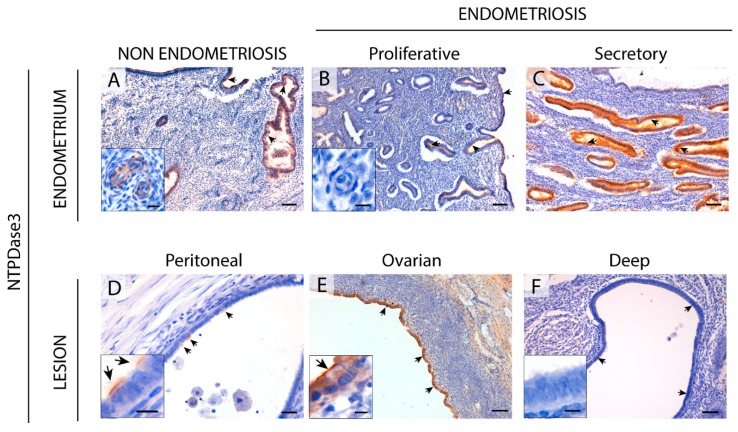
Immunolocalization of NTPDase3 in the eutopic (**A**–**C**) and ectopic (**D**–**F**) endometrial tissue. NTPDase3 was immunodetected in ciliated and non-ciliated cells of cyclic endometrium from women without (**A**) and those with (**B**,**C**) endometriosis (arrows), with changes in expression along the menstrual cycle, reaching a maximum at the secretory phase (**C**). Moreover, NTPDase3 was present in the endothelial cells of spiral arteries of women without endometriosis (inset in **A**) but not in the cyclic endometria from women with the disease (inset in **B**). In ectopic endometrial tissue, NTPDase3 was weakly expressed in the epithelial cells (arrows) of peritoneal lesions (**D**) and highly expressed in the epithelium (arrows) of the ovarian endometriomas (**E**). NTPDase3 was absent in the deep infiltrating lesions (**F**, vaginal nodule) (arrows). Insets in images (**D**–**F**) are details of the epithelium of the three different ectopic lesions. Scale bars are 100 μm (**A**–**F**), 20 μm (inset in **A**), and 10 μm (insets in **B**,**D**–**F**).

In endometrium, NTPDase3 was immunolocalized apically in the epithelial glandular cells. The expression varied during the cycle, being maximal in the secretory phase, as previously described for non-pathological endometria [8]. Ciliated cells were also apically labeled with the label accumulated at the base of the cilia (Figure 4). In addition, NTPDase3 was not detected in atrophic endometria in endometriosis (Figure S1) while it is expressed in atrophic endometria of women without the disease.

The only, but not negligible, difference between endometria from women with and those without endometriosis is the lack of NTPDase3 staining in spiral arteries in endometriosis. It was previously reported that NTPDase3 was a marker of spiral arteries, displaying a perivascular smooth muscle actin (SMA)+ labeling [8].

In endometriotic lesions, NTPDase3 expression was found in the epithelial component. And while in ovarian lesions labeling was intense, in deep infiltrating lesions labeling was sparse and it was only present in 44% of lesions (Figure 4).

### 2.4. NPP3 Expression in the Eutopic and Ectopic Endometrial Tissues

NPP3 was expressed in epithelial cells of endometria from women with endometriosis with changes in expression throughout the cycle, being maximal in the secretory phase, similar to the features described in a non-endometriosis condition [8].

**Figure 5:**
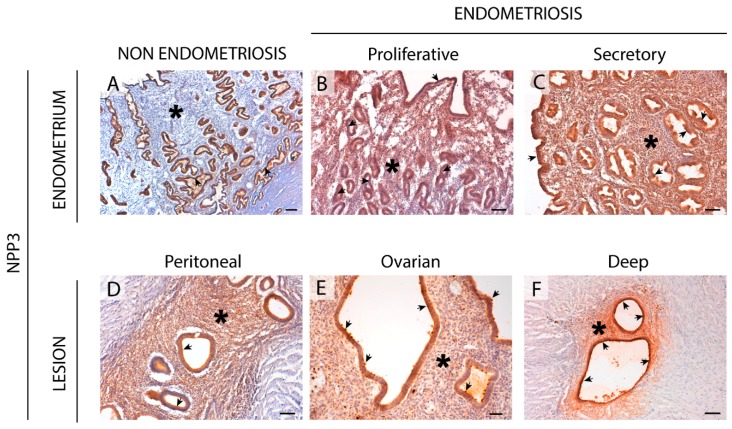
Immunolocalization of NPP3 in the eutopic (**A**–**C**) and ectopic (**D**–**F**) endometrial tissue. NPP3 is present in the luminal and glandular epithelial cells of endometrium from women without (**A**) or with (**B**,**C**) endometriosis (arrows), with changes in expression along the menstrual cycle, reaching a maximum at the secretory phase (**C**). NPP3 was expressed by the stroma (asterisks) only in endometriosis condition, including eutopic endometrium (**B**,**C**), and ectopic lesions: peritoneal (**D**), ovarian (**E**), and deep infiltrating (**F**, intestinal nodule). NPP3 is also present in the endometrial epithelial cells of lesions (arrows). Scale bars are 200 μm (**A**) and 100 μm (**B**–**F**).

Remarkably, de novo NPP3 expression was seen in the stroma of endometrial tissue, both eutopic (including atrophic) and ectopic, from women with endometriosis (Figure 5 and Figure S1). Thiamine pyrophosphatase (TPPase) in situ activity, a functional assay for E-NPPs, was also seen in the stroma of eutopic endometrium and endometriotic lesions (Figure 2).

### 2.5. CD73 Expression in the Eutopic and Ectopic Endometrial Tissues

CD73 was expressed in ciliated cells from the surface epithelium. The label is apical and comprised the entire length of the cilia. CD73 was also immunodetected in glands.

**Figure 6:**
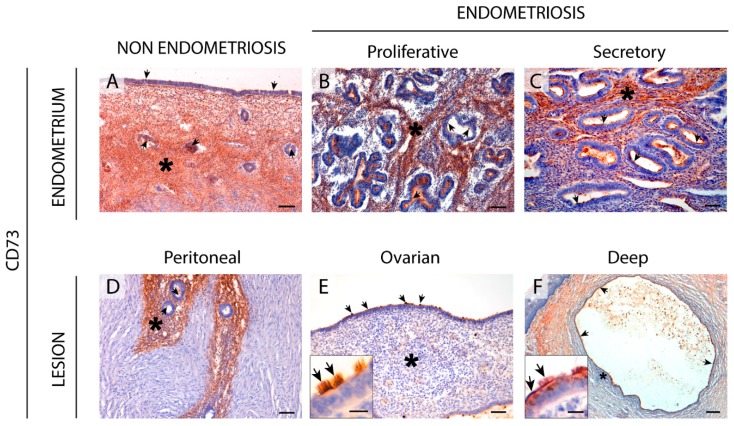
Immunolocalization of CD73 in the eutopic (**A**–**C**) and ectopic (**D**–**F**) endometrial tissue. CD73 was detected in the stroma (asterisks) of the cyclic endometrium from women without (**A**) or with (**B**,**C**) endometriosis and in the superficial peritoneal lesions (**D**). CD73 labelling was absent in the stroma (asterisks) of the ovarian (**E**) and deep infiltrating lesions (**F**, vaginal nodule). CD73 was also present in the ciliated and non-ciliated epithelial cells in the eutopic and ectopic endometrial tissue (arrows). Insets in images E and F correspond to the epithelium of ovarian and deep lesions, respectively. Scale bars are 100 μm (**A**,**B**,**D**), 50 μm (**C**,**E**), 10 μm (**E** inset), 200 μm (**F**), and 15 μm (**F** inset).

Moreover, CD73 was detected in the stromal cells in both proliferative and secretory phases, mainly in the functional layer. CD73 was, however, absent in the stromal cells of the atrophic endometria from women with endometriosis, in contrast to the atrophic endometria of women without endometriosis (Figure 6). The ectopic endometrial tissue also displayed CD73 epithelial label; however, the endometriotic lesions with stromal CD73 label decreased in relation to the severity of the lesion. In fact, 71% of peritoneal lesions presented CD73 in the endometrial stromal cells versus 48% of the ovarian endometriomas and 22% of deep infiltrating lesions.

In situ AMPase activity was detected in the same locations where CD73 was expressed and it was inhibited by the specific inhibitor α, β-methylene-ADP (α, β-meADP) (Figure 2).

### 2.6. Enzyme Dipeptidyl Peptidase IV/CD26 Expression in the Eutopic and Ectopic Endometrial Tissues

**Figure 7:**
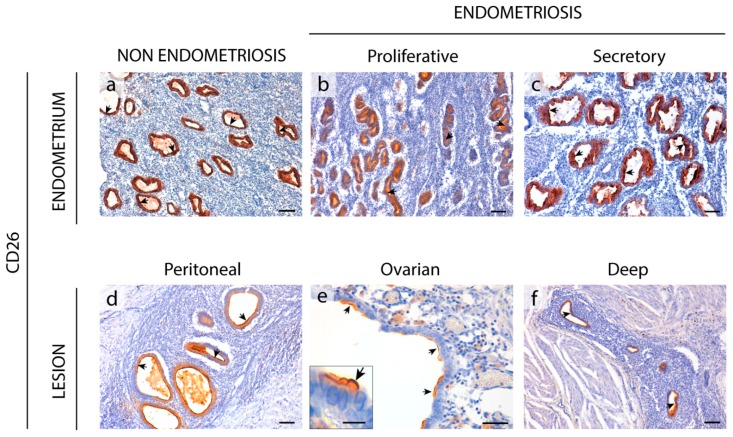
Immunolocalization of CD26 in the eutopic (**a**–**c**) and ectopic (**d**–**f**) endometrial tissue. CD26 was detected in epithelial cells (arrows) of the cyclic endometria of women without (**a**) or with (**b**,**c**) endometriosis. CD26 was also expressed by the endometrial epithelial cells (arrows) of peritoneal (**d**), ovarian (**e**), and deep infiltrating lesions (**F**, vesical nodule). Inset in (**e**) shows a detail of the CD26 labelling at the apical membrane of epithelial non-ciliated cells of an ovarian lesion. Scale bars are 100 μm (**a**–**d**,**f**), 50 μm (**e**), and 10 μm (**e** inset).

CD26 was immunodetected in endometria from women with and without endometriosis. CD26 was only detected in the epithelial cells of endometrium with the already described changes in the level of expression throughout the cycle (Figure 7). CD26 staining was very weak in atrophic endometria, and only 33% of cases presented CD26 labeling in the glandular epithelium (Figure S1).

CD26 was expressed in the epithelial component of all types of endometriotic lesions (Figure 7).

## 3. Discussion

**Figure 8:**
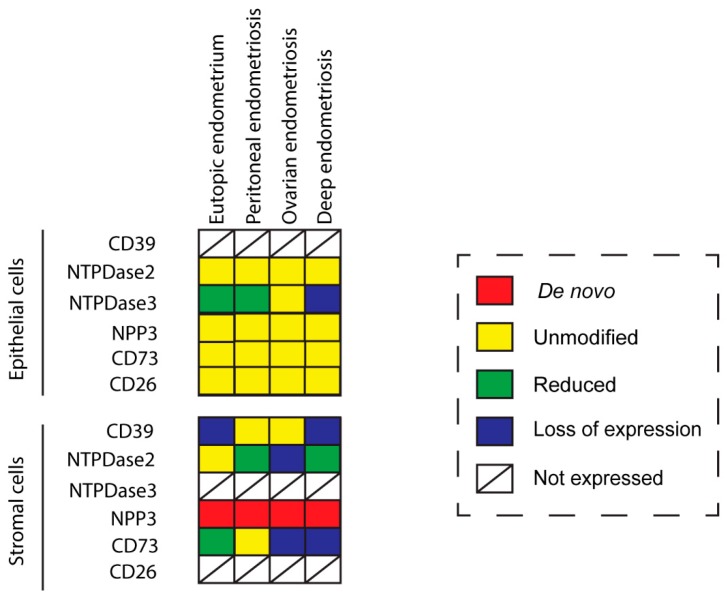
Color representation of changes of ectonucleotidase expression in the eutopic and ectopic endometrial tissue in endometriosis in comparison to the endometria of women without endometriosis. De novo, in red, indicates that this is the first time the label is detected in this cell type; unmodified; in yellow, indicates no changes in label between endometriosis and non-endometriosis; reduced, in green, indicates that the label is diminished in the endometriosis condition; loss of expression, in blue, indicates lack of expression in endometriosis; not expressed indicates that the label is never reported in this particular cell type in any condition.

Purinergic signaling plays a role in reproduction, and changes in its elements have been described in the pathology of endometriosis. Extracellular ATP may be involved in two of the major symptoms of endometriosis, which are infertility and pain [9,15,16]. In the present study we characterized the expression in eutopic and ectopic endometrial tissue of different ectonucleotidases involved in the regulation of ATP levels in tissue microenvironment. We have compared the results with those previously published in non-pathological endometria [8,14]. Tissue distribution in endometriosis coincides with the control condition except in the case of NPP3 which is present in stroma only in endometriosis. Ciliated cells of endometria display the same expression pattern as in control fallopian tubes. Changes in expression and activity are consistently recorded, the greatest being in the stroma (Figure 8). These findings provide information to elucidate the cellular and molecular mechanism as well as the etiology and the progression of the disease, which might help to identify new diagnostic and therapeutic targets.

The CD39-adenosinergic axis, with CD39 and CD73 acting sequentially to hydrolyze ATP to adenosine, is considered the main duo responsible for metabolizing extracellular ATP, generating an immunosuppressive adenosine-rich microenvironment in physiological and pathophysiological conditions [17]. In endometrium, the expression pattern of these ectonucleotidases and their changes throughout the cycle are well studied. Under physiological conditions, endometrial stromal cells express both CD39 and CD73. While CD39 expression is constant throughout the cycle, CD73 fluctuates [8,18], thus determining variations in adenosine level in the microenvironment. In the present study we note that eutopic endometrium of women with endometriosis displays the same already known expression pattern of CD73 but mostly loses CD39 stromal expression. A plausible consequence of the concomitant unbalanced ATP hydrolysis is the accumulation of extracellular ATP in the endometrial stromal microenvironment. This might well play a role in the generation and maintenance of the chronic inflammatory state of endometria of women with endometriosis. Moreover, extracellular ATP is closely related to various immune and inflammatory factors that are known to be involved in the infertility of women with endometriosis, by reducing the quality of gametes and their rates of transport and implantation, and by increasing the pregnancy loss rate [19].

This situation could explain the de novo stromal expression of NPP3 in endometriosis throughout the cycle and in atrophic endometrium, as a cellular tool to offset the loss of ATPase activity due to the lack of CD39. However, NPP3 action would not be sufficient to replace ATPase activity because NPP3 has a lower affinity for ATP than CD39 [6]; moreover stromal NPP3 expression is not coordinated with CD73 expression throughout the cycle.

Changes in the CD39-CD73 pathway were also found in endometriotic lesions. Our findings indicate that the changes in ATP hydrolysis resulting from CD39 and CD73 activity are related to the severity of endometriosis since their expression is lost in deep infiltrating lesions. These changes of expression would lead to an extracellular ATP accumulation that would in turn promote the secretion of cytokines and growth factors into the ectopic milieu, with a concomitant increase in survival and growth rates of endometrial cells [20,21]. Unfortunately, the exact function of extracellular ATP in endometriosis is not clear. While ATP signaling seems to be closely related to the origin and progression of endometriosis, intramuscular injection of ATP in a rat model of endometriosis was found to reduce the size of the ectopic induced lesions [22]. Our results are in line with previous studies that showed differing expression of the protein ATPase Na+/K+ Transporting Family Member Beta 4 (ATP1B4) between patients with and without endometriosis, in favor of a decrease of the hydrolysis of ATP in the endometriosis patients [23,24]. The authors stated that ATP was clearly related with the formation and development of endometriosis disease. Moreover, pain is a characteristic symptom of endometriosis, and ATP is a pain factor mainly acting through the purinoreceptor P2X3 that has also been studied in endometriosis. P2X3 has been found in the epithelial and some stromal cells of eutopic and ectopic endometrial tissue as well as on sensory nerve fibers in endometriotic lesions. Its expression levels correlate with the severity of pain in women with endometriosis [25]. Moreover, the use of A-317491, a selective P2X3 receptor antagonist, relieved pain with a prolonged antinociceptive effect in rats [26], and the receptor is thus a target for the pharmacological approach of endometriosis pain relief. Therefore, the increased levels of extracellular ATP might well be related to the endometriosis-associated pain. These results parallel the difference in CD73 and CD39 expression between the three entities of endometriosis, where the most extreme change has been detected in the deep infiltrating lesions, an important indicator of the severity of pain in endometriosis [27]. Additionally, the downregulation of CD73 has been described in poorly differentiated and advanced-stage endometrial carcinoma. Adenosine generated by the activity of CD73 located in the areas of cell-cell contacts regulates cell-cell adhesions by the regulation of the primary component of filopodia (F-actin). In fact, cell migration and invasion in high-grade and advanced-stage endometrial carcinomas is dependent on the loss of the adenosine generated by CD73 [28]. According to Sampson’s theory of retrograde menstruation, endometrial tissue detached during menses has to travel through the fallopian tubes to the ectopic site of implantation, such as the ovarian surface or the peritoneal wall, and must then invade and adhere to the self-tissue of the new localization, proliferating and evading the immune response to form the endometriotic lesion [29]. For this reason, the loss of CD73 in the two most severe entities of endometriosis, ovarian and deep endometriosis, as well as its implication in the inflammatory state of endometriosis, might also play a role in the migration and invasive properties of ectopic cells needed to generate the lesion.

An important finding is the identification of NPP3 label as a new histopathological marker of the disease since we have demonstrated its expression and activity in the stroma only in endometriosis, in both eutopic and ectopic tissues. NPP3 has already been identified in endometrial epithelial cells, a fact that is also confirmed in the case of endometriosis without any variation. A previous study by our group also demonstrated the presence of NPP3 in the contents of endometriomas although the levels did not differ from those of the simple ovarian cysts used as controls and therefore its presence was not exclusive of endometriosis [16]. The relevance of the study reported here is its presence in eutopic endometria which discriminates between endometriosis and non-endometriosis conditions, which might allow its use as a histopathological diagnostic tool. To our knowledge, NPP3 has been identified in epithelial cell types, in cells of the immune system, mainly mast cells, and in tumor cells with an epithelial or myeloid origin [8,30,31,32,33,34]. The specific detection of NPP3 in the endometrial stromal cells of eutopic endometria and in all three entities of endometriosis can be used as a histopathologic marker of endometriosis disease. In addition to its role in the control of extracellular ATP levels, NPP3 might well play a role in the invasive capacity of the stromal endometrial cells in endometriosis since it is known that overexpression of NPP3 in murine fibroblasts stimulates the motility and the invasiveness of these cells [35]. Our finding of de novo NPP3 in stromal cells, with greater expression in the functional layer which is shed during menses, and the relation of NPP3 with the cell motility and invasion, suggest involvement of NPP3 in the formation and progression of endometriotic lesions based on the retrograde menstruation theory [29]. Besides the importance of NPP3 as histopathological marker, additional studies are needed to determine the precise role of NPP3 in the pathogenesis and progression of endometriosis. It might well be a new target for pharmacological therapy of endometriosis. Indeed, targeting NPP3 is feasible since phase 1 trials using an antibody drug conjugate targeting this protein have been completed in patients with advanced metastatic renal cell carcinoma with promising antitumor results [36].

We found NTPDase2 expression in the same cell types and structures as in the non-endometriosis condition. Moreover, NTPDase2 was expressed by perivascular cells in some lesions with colocalization with the eMSCs marker SUSD2. Functional studies are needed to determinate whether NTPDase2+ SUSD2+ cells are eMSCs as in the eutopic endometrium. Retrograde shedding of stem cells into the pelvic cavity without immune clearance is thought to be lesion-initiating. Therefore, it would be of interest to compare lesions containing the NTPDase2+ SUSD2+ cell population with lesions without it.

NTPDase3 was described in epithelial cells and spiral arteries in healthy endometria. In fact, NTPDase3 has been considered a spiral artery marker [8]. But we did not find NTPDase3 labeling in spiral arteries of women with endometriosis. Spiral artery remodeling plays a central role in establishing and maintaining a normal pregnancy, and impaired remodeling is involved in common pregnancy disorders. This might be also one of the mechanisms underlying the decreased pregnancy rates in women with endometriosis. It is important to highlight the loss of NTPDase3 in the epithelial cells of deep infiltrating lesions. Although NTPDase3 has been little explored in pathological conditions, a decrease in *ntpdase3* expression has been described during the induction of mouse bladder cancer, suggesting its participation in cancer establishment and progression [37]. This result, together with the loss of NTPDase3 in the epithelial cells of the most severe form of endometriosis, provides further evidence of the need to study its role in the pathophysiology of endometriosis and cancer.

CD26 or dipeptidyl peptidase IV (DPPIV) is a membrane glycoprotein that binds, among other peptides, the ectoenzyme ADA in humans. It is involved in the protection of the tissue against local inflammation and in intracellular signaling. CD26 has been described as a cancer stem cell marker and tumor suppressor protein in certain types of cancer. By contrast, CD26 overexpression promotes cell proliferation, invasion, and tumorigenesis in endometrial carcinoma cells [38]. In endometriosis, Tan et al. [39] described the increase of endometrial stromal cell migration and invasion in part by reduced expression of CD26 under hypoxia conditions and also by CD26 inhibition. Other studies performed in tissue, including ours, have not matched these in vitro results with cell culture since we were not able to detect CD26 in endometrial stroma, but only in epithelial glandular cells. This might be due to the differing behavior of cells in vitro or even to technical reasons. Here, we show high expression of CD26 in the epithelial cells of eutopic endometrium and in ectopic tissue. The difference with the endometrial expression in women without endometriosis is that CD26 expression in endometriosis is constant throughout the cycle. It would be interesting to see whether the high expression of CD26 in ectopic epithelial cells has a similar effect to that of endometrial carcinoma cells on cell migration and invasion ability. In relation to the ATP metabolism, knowing the levels of ADA, the soluble enzyme that hydrolyses the extracellular adenosine to control the immunosuppressive milieu, is key to understanding what is happening in endometriosis. In a previous study, high levels of ADA were found in the contents of ovarian endometriomas [16]. We were, however, unable to detect ADA by immunostaining due to the technical limitations of the antibodies available, and we cannot be certain whether high levels of CD26 in tissue is related to an increase in ADA activity.

The changes in the expression of the ectonucleotidases described here in eutopic and ectopic endometrium argue for extracellular ATP accumulation. The greatest loss of ectonucleotidase expression was found in the deep infiltrating endometriosis, the most severe endometriosis subtype [40,41]. Our results, together with the role of ATP in pain [25,42], lend support to the involvement of ectonucleotidase expression changes with the severity of endometriosis. Moreover, our results reinforce the relevance of the stroma and tissue microenvironment in the etiopathology and progression of endometriosis disease. Future studies on the role of purinergic signaling in endometriosis are needed to identify biomarkers of the disease and to develop new therapeutic strategies that would allow for earlier detection and respect for the reproductive wishes of women with endometriosis. However, unlike in cancer, where ectonucleotidase blockade is a therapeutic tool, in endometriosis the use of inhibitors of ectonucleotidases does not seem to represent an appropriate strategy. On the contrary, increasing the ATPase activity would combat the eventual ATP accumulation of endometrial microenvironment. In line with this, the use of A-317491, an antagonist of the ATP receptor P2X3, relieves pain in endometriosis [26]. Administration of soluble CD39 is known to be safe and is well studied in the context of cardiovascular diseases where it is known to prevent thrombus formation (reviewed in [43]).

## 4. Materials and Methods

### 4.1. Samples

The ethical principles of this study adhere to the Helsinki Declaration, and all the procedures were approved by the ethics committee for clinical investigation of Bellvitge Hospital (project identification code PR090/15, Acta 21/16, 12/2016). All the patients included gave written informed consent. Fifty-seven patients with endometriotic lesions (ectopic endometrial tissue) were recruited for the study by the Gynecology Service of Bellvitge Hospital (Barcelona, Spain) between March 2016 and July 2019, and by the Gynecology Service of Dexeus Institute (Barcelona, Spain) between October 2016 and March 2018. Thirty-four endometrium samples were obtained by the Gynecology Service of Bellvitge Hospital from women without endometriosis or endometrial malignancy as a control group (including 10 proliferative, 4 secretory, and 20 atrophic endometria; age mean of patients = 55.03 years, standard derivation = 11.83).

Human endometrial samples from women with endometriosis (*n* = 25) were obtained from hysterectomy specimens without endometrial malignancy at the pathology services of Bellvitge Hospital and Dexeus Hospital. Peritoneal endometriosis (*n* = 7), ovarian endometriosis (*n* = 27), and/or deep endometriosis (*n* = 28) were surgically removed in the gynecology services of the same hospitals. Demographic description of the samples from the women with endometriosis are summarized in Table 3. Endometrial dating was carried out by the pathology services.

Excised tissue samples were fixed with 4% paraformaldehyde, cryoprotected by introducing them into a 30% (*w/v*) sucrose solution at 4 °C for 24 h, and then embedded in O.C.T freezing media (Tissue-Tek®; Sakura Finetk, Zoeterwoude, Netherlands). Fifteen μm sections were obtained using a Cryostat Leica CM1950 (Leica, Wetzlar, Germany). Sections were put onto poly-l-lysine coated glass slides and stored at −20 °C until use. Routine haematoxylin and eosin staining was performed.

### 4.2. Antibodies

Primary antibodies used in this study are listed in Table 4. Secondary antibodies used for immunohistochemistry were horseradish peroxidase (HRP)-conjugated goat anti-mouse (EnVision™ + System; DAKO, Carpinteria, CA, USA) and HRP-conjugated goat anti-rabbit (EnVision™ + System).

Secondary antibodies used for immunofluorescence assays were Alexa Fluor 488 goat anti-mouse and Alexa Fluor 647 goat anti-rabbit (Thermo Fisher Scientific, Rockford, Illinois, USA). Secondary antibodies were used at 1:500 and dilutions were made in PBS.

### 4.3. Immunolabeling Experiments

Slices were washed twice with PBS to remove the O.C.T freezing media and then pre-incubated for 1 h at room temperature (RT) with PBS containing 20% normal goat serum (NGS, Gibco, Paisley, UK), 0.2% Triton and 0.2% gelatin (Merck, Darmstadt, Germany). For immunohistochemistry experiments a previous blocking of endogenous peroxidase activity was performed with 10% methanol (*v/v*) and 2% H2O2 (*v/v*) in PBS for 30 min. Slices were then incubated overnight (O/N) at 4 °C with the primary antibodies (listed in Table 2) diluted in PBS. After three washes in PBS, tissue sections were incubated with the appropriate secondary antibodies for 1 h at RT, except HRP-goat anti-mouse and HRP-goat anti-rabbit, which were incubated for 30 min at RT. Secondary antibodies alone were routinely included as controls for the experiments.

For immunohistochemistry, the peroxidase reaction was performed in a solution containing 0.6 mg/mL 3, 3′-diaminobenzidine substrate (DAB; D-5637, Sigma-Aldrich, Saint Louis, MO, USA) and 0.5 µL/mL H2O2 in PBS for 10 min, and stopped with PBS. Nuclei were counterstained with haematoxylin and slides were then dehydrated and mounted with DPX mounting medium. Samples were observed under light Nikon Eclipse E200 and photographed under a light Leica DMD 108 microscope. In fluorescence assays, for nuclei labeling, slides were mounted with aqueous mounting medium with DAPI (ProLong™ Gold antifade reagent with DAPI, Life Technologies, Paisley, UK). Samples were then observed and photographed under a Zeiss LSM 880 Confocal Laser Scanning Microscope. Fluorescence images were processed with the software ZEN 2.3 SP1 (Zeiss, Oberkochen, Germany).

Immunohistochemical staining was independently evaluated by two observers. Staining distribution was recorded. Label intensity was scored as negative (-), weak (+), intermediate (++), or strongly positive (+++).

### 4.4. In situ ATPase, ADPase, AMPase, and TPPase Activity Experiments

A protocol based on the Wachstein/Meisel lead phosphate method was used [8,11,44,45]. The sections were washed twice with 50 mM Tris-maleate buffer pH 7.4 and pre-incubated for 30 min at RT with 50 mM Tris-maleate buffer pH 7.4 containing 2 mM MgCl_2_ and 0.25 mM sucrose. The enzymatic reaction was carried out by incubating tissue sections for 1 h at 37 °C with 50 mM Tris-maleate buffer pH 7.4 supplemented with 0.25 mM sucrose, 2 mM MgCl_2_, 5 mM MnCl_2_, 3 % Dextran, 2 mM Pb(NO_3_)_2_, and 2 mM CaCl_2_. All experiments were performed in the presence of 2.5 mM levamisole, as an inhibitor of alkaline phosphatase (AP) activity, and in the presence of 1 mM AMP, ADP, ATP, or TPP as a substrate. TPP is a false substrate, which can be cleaved by the pyrophosphatase activity of E-NPPs. Control assays were performed in the absence of nucleotide. For E-NTPDase inhibition experiments, 1 mM POM 1 was added to pre-incubation and enzymatic reaction buffers. For CD73 inhibition experiments, 1 mM α, β-meADP was added to pre-incubation and enzymatic reaction buffers. The reaction was revealed by incubation with 1% (NH_4_)_2_S (*v/v*) for exactly 1 min. Nuclei were counterstained with haematoxylin. Samples were mounted with aqueous mounting medium (FluoromountTM, Sigma-Aldrich), observed under a light Nikon Eclipse E200 microscope, and photographed under a light Leica DMD 108 microscope.

### 4.5. Statistical Analysis

The predictive analytics software IBM SPSS Statistics v22 (IBM Corp., Armonk, NY, USA) was used for the creation of frequency tables with the distribution of ectonucleotidases in each endometrial component as well as the label intensity score in each case.

## 5. Conclusions

In the present study, we examined the presence of ectonucleotidases in eutopic and ectopic endometrial tissue in endometriosis. The main changes in expression and activity were found in the stromal compartment. We observed loss of the main route of ATP hydrolysis, the CD39-CD73 axis, in deep infiltrating endometriosis, the most severe endometriosis subtype. These findings point to ATP accumulation in the endometrial tissue microenvironment in endometriosis as possibly contributing to the two main symptoms of the disease: pain and infertility. Remarkably, we noted that immunodetection of NPP3 in endometrial stroma is exclusive to the endometriosis condition, and therefore it may well be a histological marker of the disease. Future studies on the role of purinergic signaling in endometriosis are needed to elucidate the underlying cellular and molecular mechanisms and to identify new diagnostic and therapeutic targets.

## Figures and Tables

**Table 1:** Summary of the ectoenzyme expression in the eutopic endometrium from women with endometriosis.

	NTPDase1 (CD39)	NTPDase2	NTPDase3	NPP3	5′-NT (CD73)	CD26
*Proliferative endometrium*						
Surface epithelium	-	+++	+	++	++	-
Glandular epithelium						
Functional layer	-	+++	++	++	+++	++/- ^1^
Basal layer	-	+++	+	+	+++	+++
Endometrial stromal cells	-	+++	-	+++	++	-
Spiral arteries	+++	-	-	+	-	-
*Secretory endometrium*						
Surface epithelium	-	+++	+/- ^1^	+++	+++	+
Glandular epithelium						
Functional layer	-	+++	+++	+++	++	+++
Basal layer	-	+++	+++	+++	+++	++
Endometrial stromal cells	-	+++	-	+	++	-
Spiral arteries	+++	-	-	+	-	-
*Atrophic endometrium*						
Surface epithelium	-	+++	-	+	+	++/- ^1^
Glandular epithelium	-	+++	-	++	++	-
Endometrial stromal cells	++	+++	-	++	-	-
Vessels	++	-	-	+	-	-

Semi-quantitative analysis independently evaluated by two observers. Label is recorded as: (-) negative, (+) weak, (++) moderate, (+++) strong. ^1^ 50% of tissues studied had each of these staining intensities.

**Table 2:** Summary of the ectoenzyme expression in the ectopic endometrial tissue (peritoneal, ovarian, and deep infiltrative lesions) from women with endometriosis.

	NTPDase1 (CD39)	NTPDase2	NTPDase3	NPP3	5′-NT (CD73)	CD26
*Peritoneal endometriosis*						
Endometrial epithelial cells	-	+++	+	+++	+++	+++
Endometrial stromal cells	+++	++	-	++	++	-
Vessels of the lesion	+++	-	-	-	-	-
*Ovarian endometriosis*						
Endometrial epithelial cells	-	+++	+++	+++	+++	+++
Endometrial stromal cells	++	-	-	+	-	-
Vessels of the lesion	+++	-	-	-	-	-
*Deep endometriosis*						
Endometrial epithelial cells	-	+++	-	++	+++	+++
Endometrial stromal cells	-	++	-	+	-	-
Vessels of the lesion	-	-	-	-	-	-

Semi-quantitative analysis independently evaluated by two observers. Label is recorded as: (-) negative, (+) weak, (++) moderate, (+++) strong.

**Table 3:** Demographics of patients with endometriosis.

Type of Endometrium	Number of Cases	Age (years) ± Standard Deviation	Average (Range)
Proliferative	12	44.33 ± 3.11	39–51
Secretory	10	45.00 ± 5.21	38–53
Atrophic	3	43.00 ± 4.00	39–47
**Type of Lesion**			
Peritoneal	7	40.29 ± 4.96	35–46
Ovarian	27	43.42 ± 8.36	23–57
Deep	21	38.00 ± 5.73	27–47

**Table 4:** List of primary antibodies used for immunolabeling experiments.

Antibody Specificity	Name/Clone	Source	Supplier	Dilution
NTPDase1 (CD39)	BU-61	Mouse	Ancell (188-820)	1:500
NTPDase2	-	Rabbit	Enzo (ALX-215-045)	1:100
NTPDase2	H9s	Mouse	http://ectonucleotidases-ab.com	1:400
NTPDase3	B_3_S_10_	Mouse	http://ectonucleotidases-ab.com	1:500
NPP3	NP4D6	Mouse	Abcam (ab90754)	1:100
5′-nucleotidase (CD73)	4G4	Mouse	Abcam (ab81720)	1:50
CD26	202-36	Mouse	Abcam (ab3154)	1:100
CD26	202-36	Mouse	NovusBio (NBP2-44571)	1:100
SUSD2	-	Rabbit	Abcam (ab121214)	1:400

## Supplementary Materials

Supplementary materials can be found at https://www.mdpi.com/1422-0067/20/22/5532/s1. Figure S1. Immunolocalization of CD39 (A), NTPDase2 (B), NTPDase3 (C), NPP3 (D), CD73 (E), and CD26 (F) in atrophic endometria from women with endometriosis. Scale bars are 100 μm (A,C–F) and 200 μm (B). Figure S2. Immunolocalization of NTPDase2 in peritoneal (A), ovarian (B), and deep endometriosis lesions (C). There is NTPDase2 labelling in the connective tissue limiting the lesions (*arrows*). Scale bars are 100 μm (A,C) and 300 μm (B). Figure S3. Confocal fluorescence images of a proliferative endometrium (A–D) of a woman with endometriosis and an ovarian endometrioma (E–H) labeled with the antibodies against NTPDase2 (A,E) and the eMSC marker SUSD2 (B,F). Nuclei were labeled with DAPI (C,G). Merge images (D,H) show the co-localization of NTPDase2 and SUSD2 in perivascular cells in the eutopic and ectopic endometrial tissue. Scale bars are 50 μm (D,H).

## Abbreviations

ATP	Adenosine triphosphate
E-NPP	Ecto-nucleotide pyrophophotase/phosphodiesterase
E-NTPDase	Ecto-nucleoside triphosphate diphosphohydrolase
ADP	Adenosine diphosphate
AMP	Adenosine monophosphate
5′-NT	5′-nucleotidase
ADA	Adenosine deaminase
eMSC	Endometrial mesenchymal stem cell
SUSD2	Sushi domain containing 2
SMA	Smooth muscle actin
TPP	Thiamine pyrophosphate
α, β-meADP	α, β-methylene-ADP
ATP1B4	ATPase Na+/K+ Transporting Family Member Beta 4
DPPIV	Dipeptidyl peptidase IV
HRP	Horseradish peroxidase
DAB	3, 3′-diaminobenzidine substrate
AP	Alkaline phosphatase

## Appendix A

**Table A1:** Summary of the number and proportion of tissues labeled with different antibodies.

Samples Stained	NTPDase1 (CD39)	NTPDase2	NTPDase3	NPP3	5′-NT (CD73)	CD26
	Positive Cases/Total of Samples (% of Tissues Stained)					
***Eutopic endometrium***						
Surface epithelium	1/14 (7.1%)	14/14 (100%)	7/13 (53.8%)	12/13 (92.3%)	9/12 (75%)	7/14 (50%)
Glandular epithelium	1/20 (5%)	20/20 (100%)	16/20 (80%)	18/19 (94.7%)	20/20 (100%)	15/20 (75%)
Endometrial stromal cells	7/20 (35%)	20/20 (100%)	0/20 (0%)	15/19 (78.9%)	12/20 (60%)	0/20 (0%)
Spiral arteries	19/20 (95%)	3/20 (15%)	5/20 (25%)	16/18 (88.9%)	3/20 (15%)	3/19 (15.8%)
***Peritoneal endometriosis***						
Endometrial epithelial cells	1/7 (14.3%)	7/7 (100%)	7/7 (100%)	7/7 (100%)	5/7 (71.4%)	4/7 (57.1%)
Endometrial stromal cells	6/7 (85.7%)	7/7 (100%)	0/7 (0%)	7/7 (100%)	5/7 (71.4%)	2/7 (28.6%)
Vessels of the lesion	5/7 (71.4%)	0/7 (0%)	0/7 (0%)	0/7 (0%)	1/7 (14.28%)	0/7 (0%)
***Ovarian endometriosis***						
Endometrial epithelial cells	8/28 (28.6%)	20/28 (71.4%)	16/28 (57.1%)	24/28 (85.7%)	24/28 (85.7%)	19/28 (67.9%)
Endometrial stromal cells	16/27 (59.3%)	10/27 (37%)	3/27 (11.1%)	16/27 (59.3%)	13/27 (48.1%)	2/27 (7.4%)
Vessels of the lesion	23/28 (82.1%)	1/28 (3.6%)	2/28 (7.1%)	8/28 (28.6%)	3/28 (10.7%)	3/28 (10.7%)
***Deep endometriosis***						
Endometrial epithelial cells	6/28 (21.4%)	23/26 (88.5%)	12/27 (44.4%)	24/28 (85.7%)	17/28 (60.7%)	15/28 (53.6%)
Endometrial stromal cells	9/25 (36%)	17/26 (65.4%)	1/27 (3.7%)	18/27 (66.7%)	6/27 (22.2%)	1/27 (3.7%)
Vessels of the lesion	11/24 (45.8%)	5/25 (20%)	1/27 (3.7%)	7/27 (25.9%)	7/27 (25.9%)	0/27 (0%)
