# Supplementary figures and images for: Impaired Expression of Ectonucleotidases in Ectopic and Eutopic Endometrial Tissue Is in Favor of ATP Accumulation in the Tissue Microenvironment in Endometriosis
Source: Int J Mol Sci. 2019 Nov 6;20(22):5532. doi: 10.3390/ijms20225532 (PMC6888134; doi:10.3390/ijms20225532)

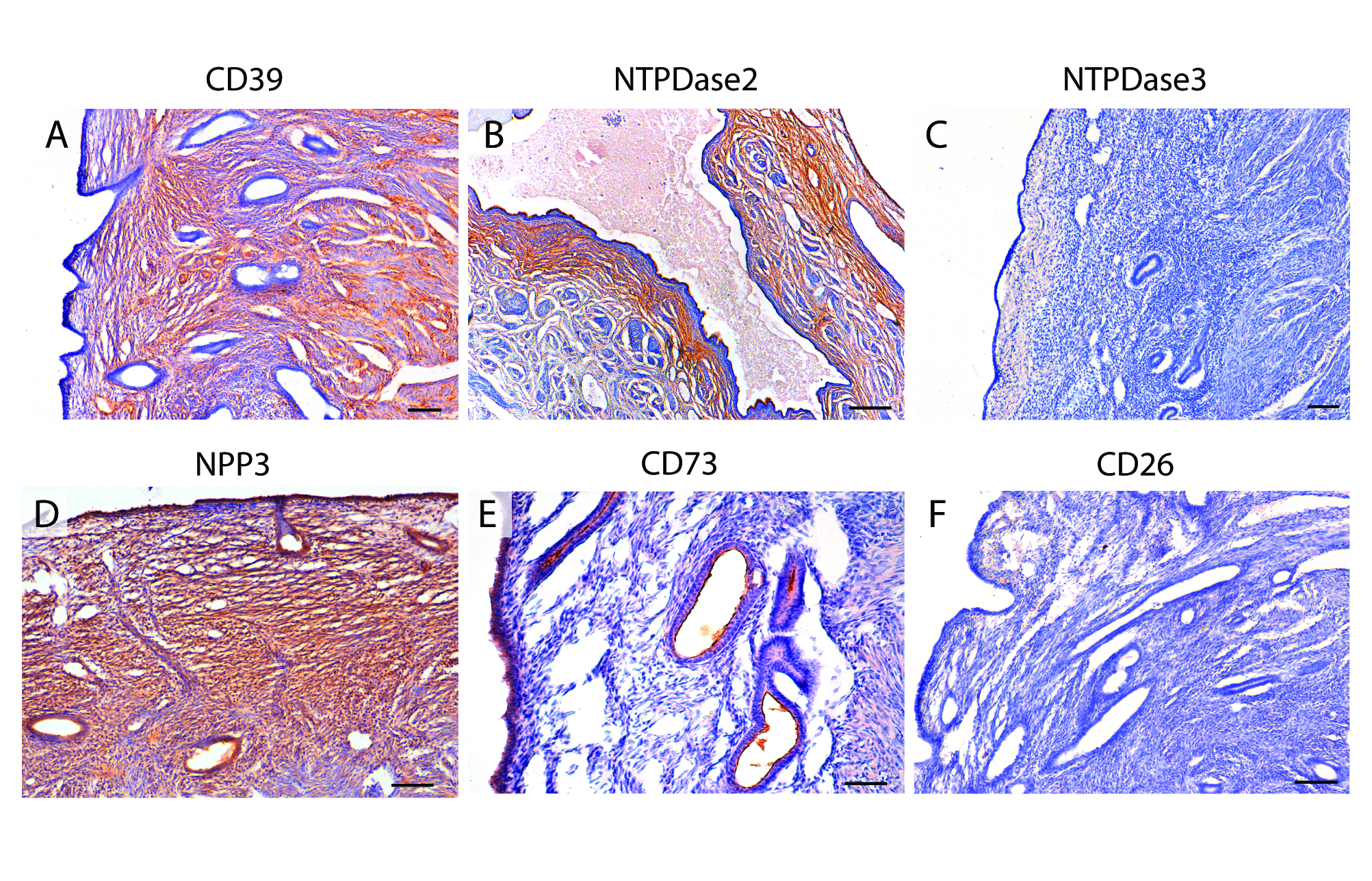

Supplement: Supplementary file 1 [file ijms-20-05532-s001.zip › Figure S1_Trapero_et_al_2019.tif]

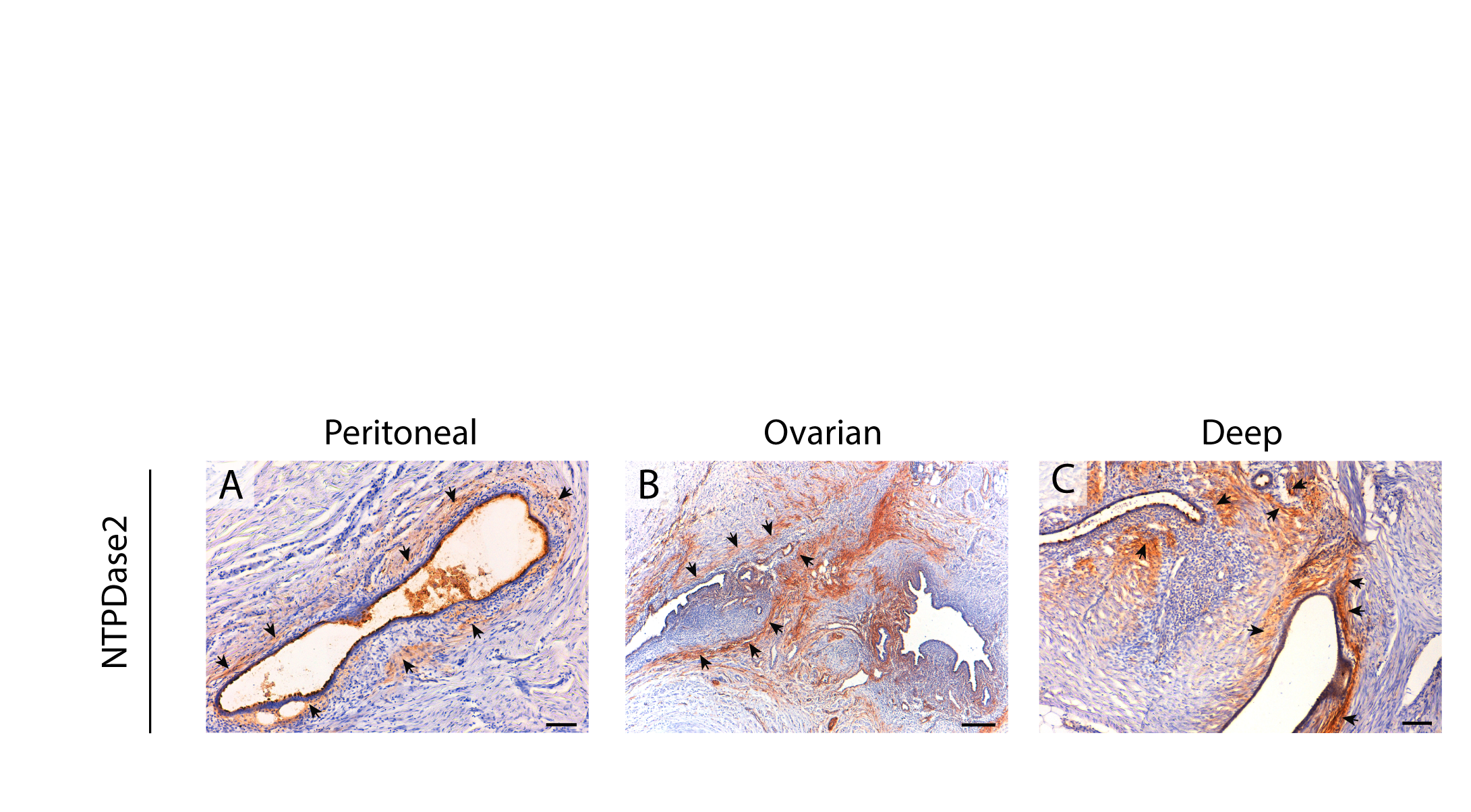

Supplement: Supplementary file 1 [file ijms-20-05532-s001.zip › Figure S2_Trapero_et_al_2019.tif]

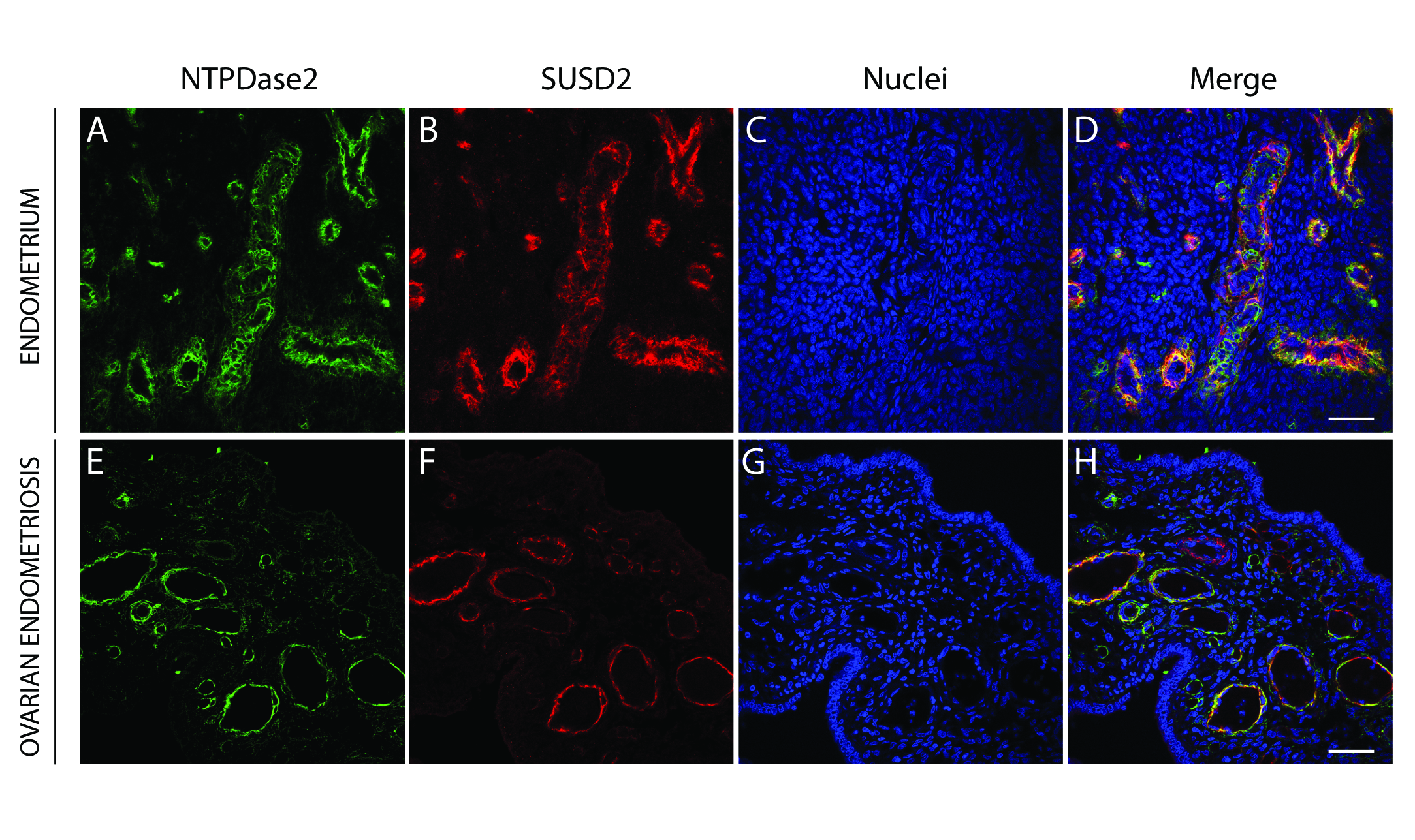

Supplement: Supplementary file 1 [file ijms-20-05532-s001.zip › Figure S3_Trapero_et_al_2019.tif]
